# Supplementary material for: Peri active site catalysis of proline isomerisation is the molecular basis of allomorphy in β-phosphoglucomutase
Source: Commun Biol. 2024 Jul 27;7:909. doi: 10.1038/s42003-024-06577-9 (PMC11283535; doi:10.1038/s42003-024-06577-9)
Supplement: Supplementary file 2 — Supplementary Information [file 42003_2024_6577_MOESM2_ESM.pdf]

SUPPLEMENTARY INFORMATION

Peri active site catalysis of proline isomerisation is the molecular basis of allomorphy in β-phosphoglucomutase

F. Aaron Cruz-Navarrete[1,3,#], Nicola J. Baxter[1,2,#], Adam J. Flinders[1], Anamaria Buzoianu[1,4], Matthew J. Cliff[2], Patrick J. Baker[1] & Jonathan P. Waltho[1,2,*]

[1] School of Biosciences, University of Sheffield, Sheffield, S10 2TN, United Kingdom.

[2] Manchester Institute of Biotechnology and Department of Chemistry, The University of Manchester, Manchester, M1 7DN, United Kingdom.

[3] Present address: Department of Structural Biology, St. Jude Children's Research Hospital, Memphis, Tennessee, 38105, USA.

[4] Present address: Department of Chemistry, Biochemistry and Pharmacy, University of Bern, Bern, 3012, Switzerland.

[#] F.A.C.N. and N.J.B. contributed equally

* To whom correspondence may be addressed:

Prof. Jonathan P. Waltho, School of Biosciences, University of Sheffield, Sheffield, S10 2TN, United Kingdom, +44 114 2222717, j.waltho@sheffield.ac.uk, ORCID 0000-0002-7402-5492.

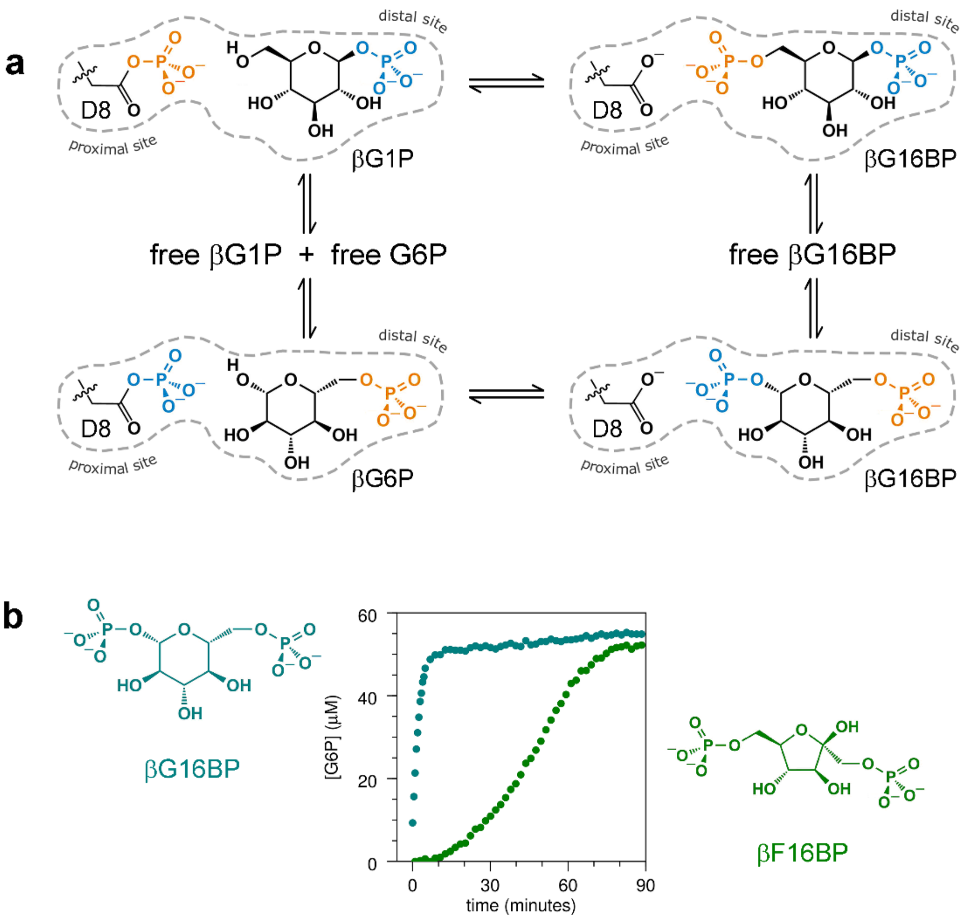

Supplementary Figure 1 | βPGM catalytic cycle and reaction kinetics. **a** βPGM catalytic cycle describing the enzymatic conversion of βG1P to G6P via a βG16BP reaction intermediate. The phosphoryl transfer reaction between phosphorylated βPGM (βPGM^P, phosphorylated at residue D8) and βG1P is shown with the transferring phosphate (orange) in the proximal site and the phosphate group (blue) of βG1P in the distal site. The βG16BP product is released to solution, which rebinds in the alternative orientation[1]. The phosphoryl transfer reaction between βPGM and βG16BP is illustrated with the transferring phosphate (blue) in the proximal site and the 6-phosphate group (orange) of βG16BP in the distal site. G6P is released as a product along with the regeneration of βPGM^P. **b** Reaction kinetics for the conversion of βG1P to G6P using different allomorphic activators (phosphorylating agents) to generate βPGM^P. The rate of G6P production was measured indirectly using a glucose 6-phosphate dehydrogenase (G6PDH) coupled assay, in which G6P is oxidised and concomitant NAD^+ reduction is monitored by the increase in absorbance at 340 nm. The reaction was catalysed by βPGM_{WT} using either βG16BP (teal circles, $k_{cat} = 382 \pm 12\ s^{-1}$) or F16BP (green circles) as an allomorphic activator. Reaction conditions: 5 nM βPGM_{WT}, 50 μM βG1P and either 10 μM βG16BP or 1 mM F16BP were added to solutions containing 1 mM NAD^+, 5 units mL^{-1} G6PDH, 200 mM HEPES (pH 7.2), 5 mM $MgCl_2$ and 1 mM NaN_3 [2]. For clarity, not all of the acquired data points have been included in the kinetic profiles. In solution, F16BP forms an equilibrium mixture of αF16BP (15 %), βF16BP (81 %) and two open chain forms with an interconversion rate of 8 s^{-1} [3]. βF16BP is the biologically active anomer and the chemical structures of βG16BP and βF16BP are shown for comparison.

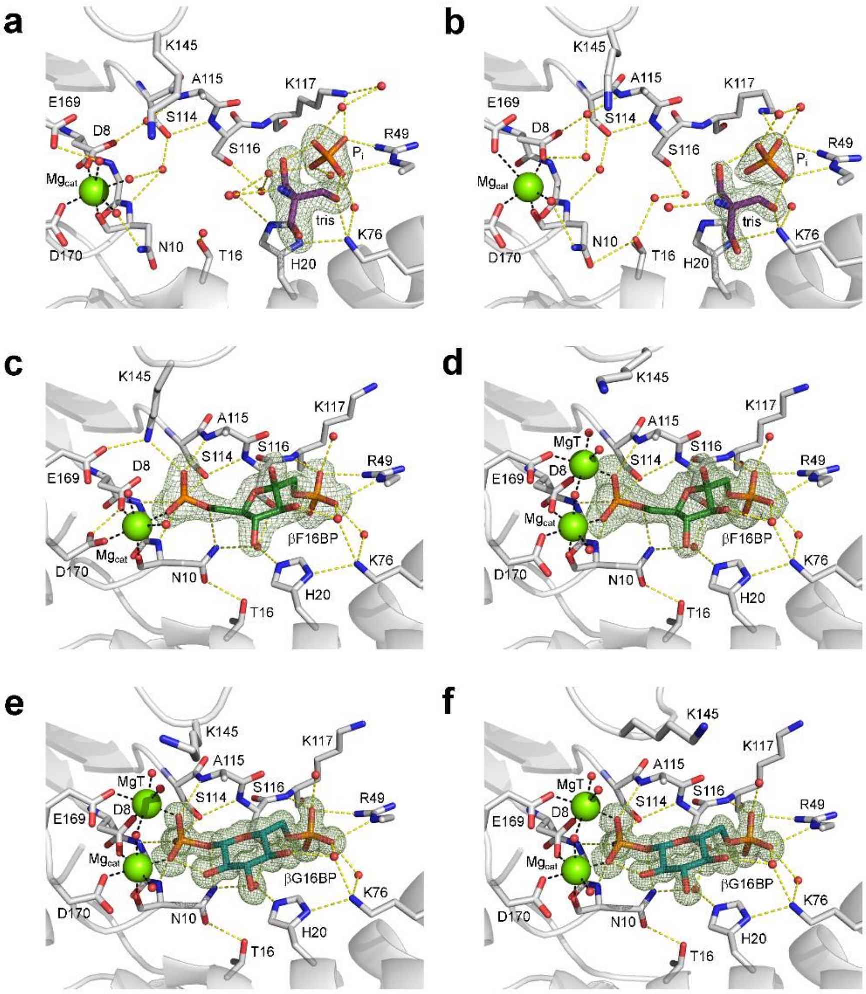

Supplementary Figure 2 | Difference density and active site details of substrate-free βPGM$_{D10N,P146A}$ and βPGM complexes. The active site of **a** substrate-free *trans*-A βPGM$_{D10N,P146A}$ (PDB 8Q1C chain A), **b** substrate-free *trans*-A βPGM$_{D10N,P146A}$ (PDB 8Q1C chain B), **c** the *cis*-P βPGM$_{D10N}$:F16BP complex (PDB 8Q1D), **d** the *trans*-A βPGM$_{D10N,P146A}$:F16BP:MgT complex (PDB 8Q1E), **e** the *trans*-A βPGM$_{D10N,P146A}$:βG16BP:MgT complex (PDB 8Q1F chain A) and **f** the *trans*-A βPGM$_{D10N,P146A}$:βG16BP:MgT complex (PDB 8Q1F chain B). Selected residues (sticks), together with P_i, tris (purple carbon atoms), F16BP (dark green carbon atoms), βG16BP (teal carbon atoms), structural waters (red spheres), Mg_{cat} (green sphere) and MgT (green sphere) are illustrated. Yellow dashes indicate hydrogen bonds and black dashes show metal ion coordination. Difference density (Fo – Fc, green mesh) is contoured at 3σ and was generated following ligand omission from the final structures. Apart from R49, residues of the substrate specificity loop (V36–L53) have been omitted for clarity.

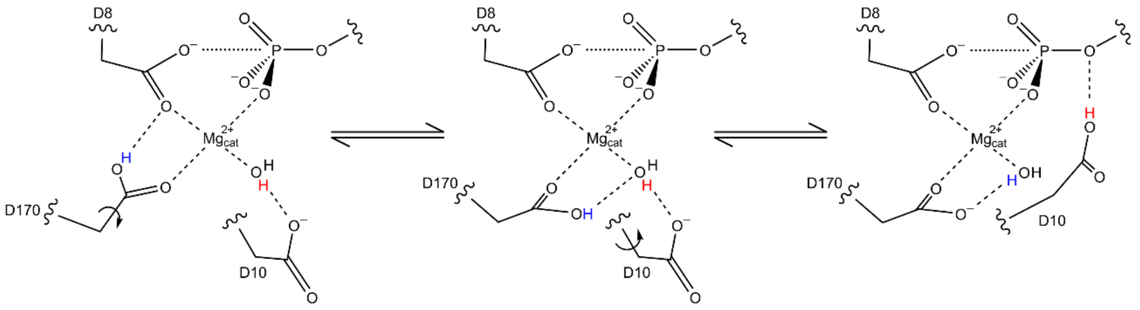

Supplementary Figure 3 | Schematic representation of the proton shuttling pathway model operating in the active site of βPGM that assists protonation of the general acid-base. In NAC conformations, the phosphodianion group of either F16BP or βG16BP in the proximal site is located in an anion-rich environment. Protonation of the carboxylate sidechain of D170 (blue H) in a NAC I conformation, corroborated by the close proximity of the sidechains of D8 and D170 in the *cis*-P βPGM$_{D10N}$:F16BP complex (PDB 8Q1D), would offer more complete charge neutralisation. During the structural transition from the NAC I conformation towards the NAC III conformation, a sidechain rotation of D170 could deliver a second proton (red H) to the nascent leaving group via a water molecule coordinating Mg$_{cat}$ along with the carboxylate sidechain of D10 on its positional switch from a solvent exposed location to one within the active site. Hydrogen bonds and metal ion coordination are shown by dashed lines and the aligned phosphorus-nucleophilic oxygen donor-acceptor relationship is indicated by a dotted line.

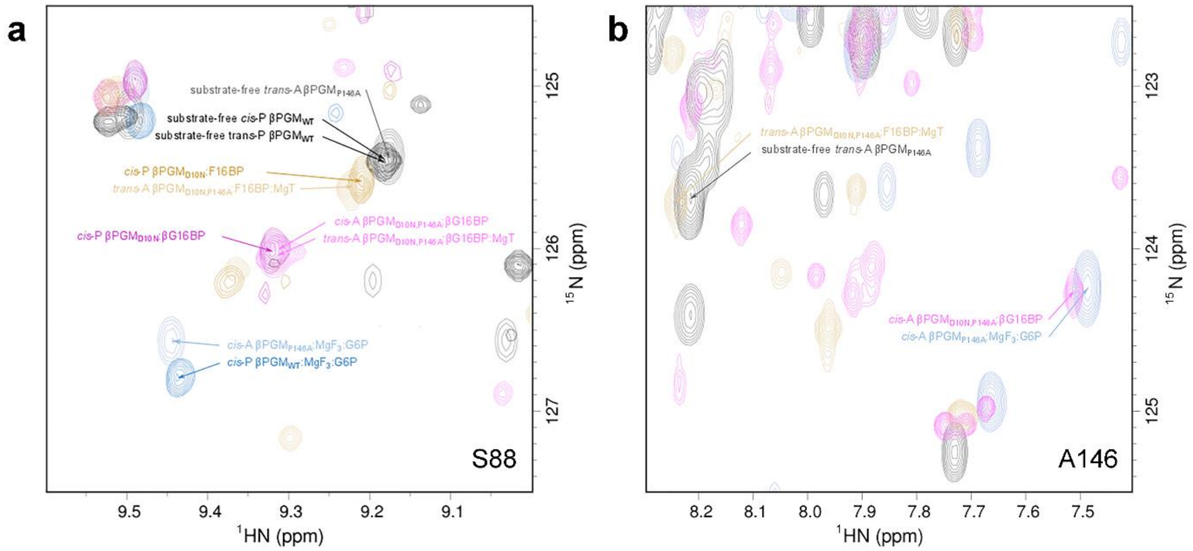

Supplementary Figure 4 | Overlays of $^1H^{15}N$-TROSY spectra highlighting the behaviour of residues S88 and A146 in substrate-free βPGM species and βPGM complexes. **a**, **b** The corresponding peak positions for either S88 or A146 in each of the $^1H^{15}N$-TROSY spectra are labelled accordingly. **a** S88 is a reporter of the interdomain hinge closure angle. The open conformation of substrate-free *cis*-P βPGM$_{WT}$ (black), substrate-free *trans*-P βPGM$_{WT}$ (black) and substrate-free *trans*-A βPGM$_{P146A}$ (grey), along with the NAC I conformation of *cis*-P βPGM$_{D10N}$:F16BP (gold) and *trans*-A βPGM$_{D10N,P146A}$:F16BP:MgT (beige), and the NAC III conformation of *cis*-P βPGM$_{D10N}$:βG16BP (purple), *cis*-A βPGM$_{D10N,P146A}$:βG16BP (pink) and *trans*-A βPGM$_{D10N,P146A}$:βG16BP:MgT (pink) describe a transition in both backbone amide ^{1}HN and ^{15}N chemical shifts towards the fully closed near-transition state conformation of *cis*-P βPGM$_{WT}$:MgF$_3$:G6P (blue) and *cis*-A βPGM$_{P146A}$:MgF$_3$:G6P (pale blue). **b** A146 is sensitive to the isomerisation state of the K145-A146 peptide bond and clusters either at ^{1}HN ~ 8.2 ppm in substrate-free *trans*-A βPGM$_{P146A}$ (grey) and *trans*-A βPGM$_{D10N,P146A}$:F16BP:MgT (beige) or at ^{1}HN ~ 7.5 ppm in *cis*-A βPGM$_{P146A}$:MgF$_3$:G6P (pale blue) and *cis*-A βPGM$_{D10N,P146A}$:βG16BP (pink).

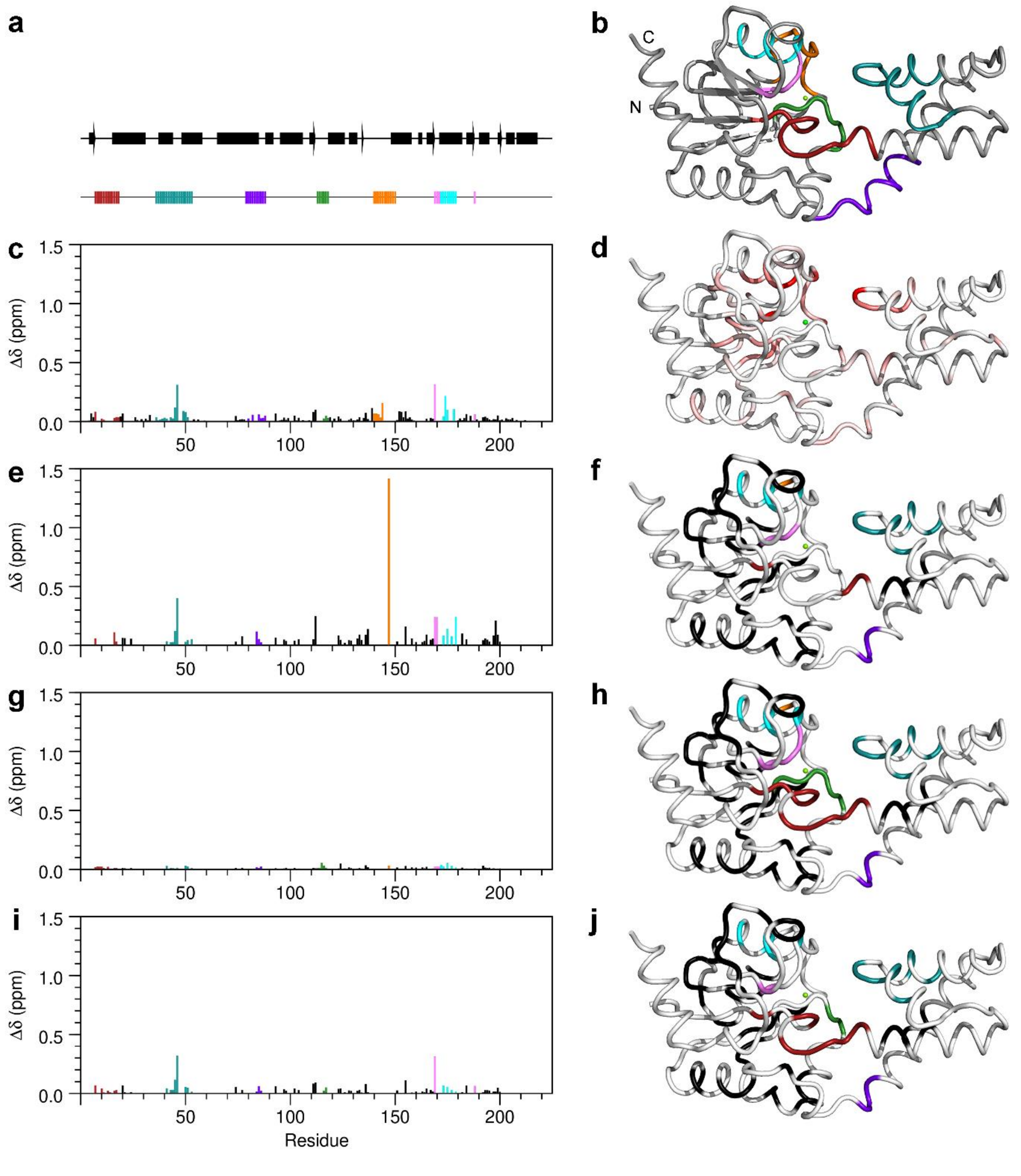

Supplementary Figure 5 | Weighted chemical shift perturbations reporting differences in the solution conformations of βPGM:F16BP complexes. **a**, **b** Scheme showing the architecture of substrate-free *cis*-P βPGM_{WT} (PDB 2WHE[6]) with secondary structure elements indicated by bars (α-helices) and arrows (β-strands). Active site regions are highlighted by coloured bars and coloured cartoon backbone: general acid-base hinge (dark red, F7–E18), substrate specificity loop (teal, V36–L53), 80s hinge (purple, N79–S88), phosphodianions bridging loop (green, A113–N118), allomorphic control loop (orange, E140–I150), Mg_{cat} site (pink, E169–S171, V188) and 170s α-helix (cyan, Q172–K179). **c**–**h** Weighted chemical shift perturbations of the backbone amide group are calculated for each residue as: $\Delta\delta = [(\delta_{HN\text{-}X} - \delta_{HN\text{-}Y})^2 + (0.13 \times (\delta_{N\text{-}X} - \delta_{N\text{-}Y}))^2]^{1/2}$, where X and Y are the two βPGM complexes being compared. Δδ values are shown by coloured histogram bars and red-shaded cartoon

backbone. **c**, **d** Δδ values between the *trans*-A βPGM$_{D10N,P146A}$:F16BP:MgT complex (BMRB 51986) and the *trans*-A βPGM$_{D10N,P146A}$:F16BP complex (BMRB 51987). The Δδ values indicate that there is a small but general propagation of effects observed throughout the structure on MgT binding in a NAC I conformation. **e–j** Δδ values between pairs of βPGM complexes are shown by histogram bars and residue locations are indicated by either a coloured or black cartoon backbone. **e**, **f** Δδ values between the *trans*-P βPGM$_{D10N}$:F16BP:MgT complex and the *cis*-P βPGM$_{D10N}$:F16BP complex (BMRB 51985) (pairwise comparison comprises 62 residues). The Δδ values reflect the impact of both the different isomerisation state of the K145-A146 peptide bond and the occupancy of MgT in the active site. **g**, **h** Δδ values between the *trans*-P βPGM$_{D10N}$:F16BP:MgT complex and the *trans*-A βPGM$_{D10N,P146A}$:F16BP:MgT complex (BMRB 51986) (pairwise comparison comprises 77 residues). Broadly distributed residues exhibit near-negligible Δδ values, indicating that these complexes have virtually identical solution behaviour, which confirms the identity of the *trans*-P βPGM$_{D10N}$:F16BP:MgT complex. **i**, **j** Δδ values between the *trans*-P βPGM$_{D10N}$:F16BP:MgT complex and the *trans*-A βPGM$_{D10N,P146A}$:F16BP complex (BMRB 51987) (pairwise comparison comprises 68 residues). Small Δδ values reflect the differential occupancy of MgT.

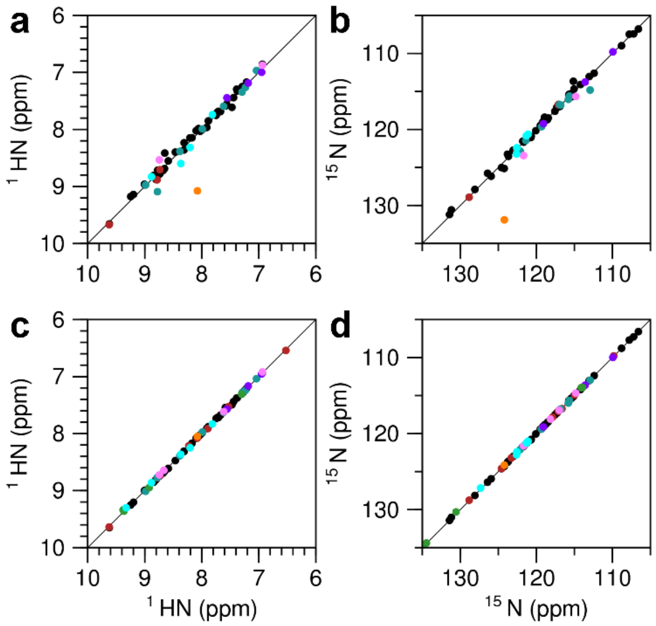

Supplementary Figure 6 | Backbone amide chemical shift correlations between pairs of βPGM:F16BP complexes. **a**, **b** Comparison between the *trans*-P βPGM_{D10N}:F16BP:MgT complex (x-axis) and the *cis*-P βPGM_{D10N}:F16BP complex (y-axis, BMRB 51985) for **a** the backbone amide ^{1}HN chemical shifts and **b** the backbone amide ^{15}N chemical shifts. The Pearson correlation coefficients are 0.9778 and 0.9807, respectively. **c**, **d** Comparison between the *trans*-P βPGM_{D10N}:F16BP:MgT complex (x-axis) and the *trans*-A $\beta PGM_{D10N,P146A}$:F16BP:MgT complex (y-axis, BMRB 51986) for **c** the backbone amide ^{1}HN chemical shifts and **d** the backbone amide ^{15}N chemical shifts. The Pearson correlation coefficients are 0.9998 and 0.9999, respectively. Residue specific chemical shift comparisons are indicated by filled circles and coloured according to the βPGM structural regions that are defined in Fig. 7 and Supplementary Fig. 5. Due to its close proximity, residue A147 (orange) is a sensitive reporter of the isomerisation state of the K145-X146 peptide bond. A z-test on Fisher z-transformed correlation coefficients was performed to determine the statistical significance of the difference between the two independent correlation coefficients. For both the ^{1}HN and ^{15}N chemical shift correlations, the P-value derived is less than 0.05, which indicates that the correlation coefficients are significantly different. Taken together, this analysis confirms the identity of the *trans*-P βPGM_{D10N}:F16BP:MgT complex.

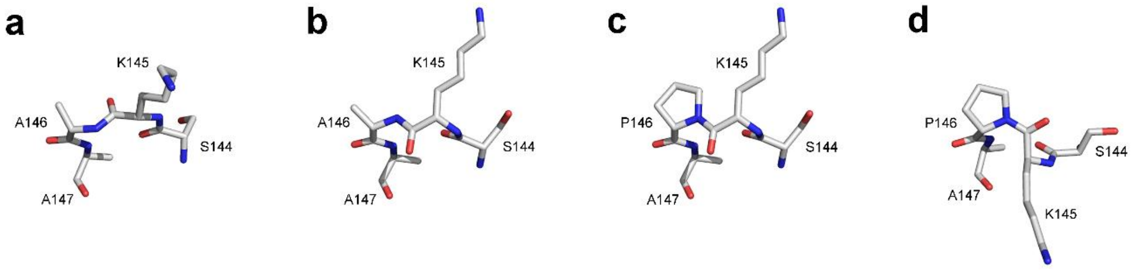

Supplementary Figure 7 | A model of the orientation of a *trans* K145-P146 peptide bond can be proposed using the geometry of a *trans* K145-A146 peptide bond. **a**–**d** Conformational arrangement of residues S144–A147 within the allomorphic control loop. **a** Geometry of the *trans* K145-A146 peptide bond in substrate-free *trans*-A βPGM$_{P146A}$ (PDB 6YDK[2]). The observed rotamer of the K145-A146 peptide bond precludes the required bonding arrangement for the pyrrolidine ring of proline. **b** Geometry of the *trans* K145-A146 peptide bond in the *trans*-A βPGM$_{D10N,P146A}$:βG16BP:MgT complex (PDB 8Q1F, chain B). **c** Model of a *trans* K145-P146 peptide bond adapted from the *trans*-A βPGM$_{D10N,P146A}$:βG16BP:MgT complex (PDB 8Q1F, chain B). **d** Geometry of the *cis* K145-P146 peptide bond in the *cis*-P βPGM$_{D10N}$:βG16BP complex (PDB 5OK1[5]). **b**, **d** The correspondence in geometry between the *trans* K145-A146 peptide bond shown in **b** and the *cis* K145-P146 peptide bond depicted in **d** provides an appropriate model for the conformation of a *trans* K145-P146 peptide bond illustrated in **c**. This correspondence includes: alignment between the N–H bond of A146 and the N–Cδ bond of P146, alignment between the Cα–Cβ bond of A146 and the Cα–Cβ bond of P146, and a similar positional relationship of the Cα and C' atoms of K145 in the *trans* K145-A146 peptide bond and the C' and Cα atoms of K145 in the *cis* K145-P146 peptide bond.

········10·························38····44······53·····································96··················117························145·····················170···

Substrate-free *cis*-P βPGM$_{WT}$ (BMRB 28095[2])

MFKAVLFDLDGVITDTAEYHFRAWKALAEEIGINGVDRQFNEQLKGVSREDSLQKILDLADKKVSAEEFKELAKRKNDNYVKMIQDVSPADVYPGILQLLKDLRSNKIKIALASASKNGPFLLERMNLTGYFDAIADPAEVAASKPAPDIFIAAAHAVGVAPSESIGLEDSQAGIQAIKDSGALPIGVGRPEDLGDDIVIPDTSHYTLEFLKEVWLQKQK

Substrate-free *trans*-P βPGM$_{WT}$ (BMRB 28096[2])

MFKAVLFDLDGVITDTAEYHFRAWKALAEEIGINGVDRQFNEQLKGVSREDSLQKILDLADKKVSAEEFKELAKRKNDNYVKMIQDVSPADVYPGILQLLKDLRSNKIKIALASASKNGPFLLERMNLTGYFDAIADPAEVAASKPAPDIFIAAAHAVGVAPSESIGLEDSQAGIQAIKDSGALPIGVGRPEDLGDDIVIPDTSHYTLEFLKEVWLQKQK

Substrate-free *cis*-P βPGM$_{D10N}$

MFKAVLFDLNGVITDTAEYHFRAWKALAEEIGINGVDRQFNEQLKGVSREDSLQKILDLADKKVSAEEFKELAKRKNDNYVKMIQDVSPADVYPGILQLLKDLRSNKIKIALASASKNGPFLLERMNLTGYFDAIADPAEVAASKPAPDIFIAAAHAVGVAPSESIGLEDSQAGIQAIKDSGALPIGVGRPEDLGDDIVIPDTSHYTLEFLKEVWLQKQK

Substrate-free *trans*-P βPGM$_{D10N}$

MFKAVLFDLNGVITDTAEYHFRAWKALAEEIGINGVDRQFNEQLKGVSREDSLQKILDLADKKVSAEEFKELAKRKNDNYVKMIQDVSPADVYPGILQLLKDLRSNKIKIALASASKNGPFLLERMNLTGYFDAIADPAEVAASKPAPDIFIAAAHAVGVAPSESIGLEDSQAGIQAIKDSGALPIGVGRPEDLGDDIVIPDTSHYTLEFLKEVWLQKQK

Substrate-free *trans*-A βPGM$_{P146A}$ (BMRB 27920[4])

MFKAVLFDLDGVITDTAEYHFRAWKALAEEIGINGVDRQFNEQLKGVSREDSLQKILDLADKKVSAEEFKELAKRKNDNYVKMIQDVSPADVYPGILQLLKDLRSNKIKIALASASKNGPFLLERMNLTGYFDAIADPAEVAASKAAPDIFIAAAHAVGVAPSESIGLEDSQAGIQAIKDSGALPIGVGRPEDLGDDIVIPDTSHYTLEFLKEVWLQKQK

Substrate-free *trans*-A βPGM$_{D10N,P146A}$

MFKAVLFDLNGVITDTAEYHFRAWKALAEEIGINGVDRQFNEQLKGVSREDSLQKILDLADKKVSAEEFKELAKRKNDNYVKMIQDVSPADVYPGILQLLKDLRSNKIKIALASASKNGPFLLERMNLTGYFDAIADPAEVAASKAAPDIFIAAAHAVGVAPSESIGLEDSQAGIQAIKDSGALPIGVGRPEDLGDDIVIPDTSHYTLEFLKEVWLQKQK

cis-P βPGM$_{D10N}$:F16BP complex (BMRB 51985)

MFKAVLFDLNGVITDTAEYHFRAWKALAEEIGINGVDRQFNEQLKGVSREDSLQKILDLADKKVSAEEFKELAKRKNDNYVKMIQDVSPADVYPGILQLLKDLRSNKIKIALASASKNGPFLLERMNLTGYFDAIADPAEVAASKPAPDIFIAAAHAVGVAPSESIGLEDSQAGIQAIKDSGALPIGVGRPEDLGDDIVIPDTSHYTLEFLKEVWLQKQK

trans-A βPGM$_{D10N,P146A}$:F16BP complex (BMRB 51987)

MFKAVLFDLNGVITDTAEYHFRAWKALAEEIGINGVDRQFNEQLKGVSREDSLQKILDLADKKVSAEEFKELAKRKNDNYVKMIQDVSPADVYPGILQLLKDLRSNKIKIALASASKNGPFLLERMNLTGYFDAIADPAEVAASKAAPDIFIAAAHAVGVAPSESIGLEDSQAGIQAIKDSGALPIGVGRPEDLGDDIVIPDTSHYTLEFLKEVWLQKQK

trans-A βPGM$_{D10N,P146A}$:F16BP:MgT complex (BMRB 51986)

MFKAVLFDLNGVITDTAEYHFRAWKALAEEIGINGVDRQFNEQLKGVSREDSLQKILDLADKKVSAEEFKELAKRKNDNYVKMIQDVSPADVYPGILQLLKDLRSNKIKIALASASKNGPFLLERMNLTGYFDAIADPAEVAASKAAPDIFIAAAHAVGVAPSESIGLEDSQAGIQAIKDSGALPIGVGRPEDLGDDIVIPDTSHYTLEFLKEVWLQKQK

cis-P βPGM$_{D10N}$:βG16BP complex (BMRB 27174[5])

MFKAVLFDLNGVITDTAEYHFRAWKALAEEIGINGVDRQFNEQLKGVSREDSLQKILDLADKKVSAEEFKELAKRKNDNYVKMIQDVSPADVYPGILQLLKDLRSNKIKIALASASKNGPFLLERMNLTGYFDAIADPAEVAASKPAPDIFIAAAHAVGVAPSESIGLEDSQAGIQAIKDSGALPIGVGRPEDLGDDIVIPDTSHYTLEFLKEVWLQKQK

cis-P Mg$_{cat}$-free βPGM$_{D10N}$:βG16BP complex (BMRB 27175[5])

MFKAVLFDLNGVITDTAEYHFRAWKALAEEIGINGVDRQFNEQLKGVSREDSLQKILDLADKKVSAEEFKELAKRKNDNYVKMIQDVSPADVYPGILQLLKDLRSNKIKIALASASKNGPFLLERMNLTGYFDAIADPAEVAASKPAPDIFIAAAHAVGVAPSESIGLEDSQAGIQAIKDSGALPIGVGRPEDLGDDIVIPDTSHYTLEFLKEVWLQKQK

cis-A βPGM$_{D10N,P146A}$:βG16BP complex (BMRB 51989)

MFKAVLFDLNGVITDTAEYHFRAWKALAEEIGINGVDRQFNEQLKGVSREDSLQKILDLADKKVSAEEFKELAKRKNDNYVKMIQDVSPADVYPGILQLLKDLRSNKIKIALASASKNGPFLLERMNLTGYFDAIADPAEVAASKAAPDIFIAAAHAVGVAPSESIGLEDSQAGIQAIKDSGALPIGVGRPEDLGDDIVIPDTSHYTLEFLKEVWLQKQK

trans-A βPGM$_{D10N,P146A}$:βG16BP complex (BMRB 51990)

MFKAVLFDLNGVITDTAEYHFRAWKALAEEIGINGVDRQFNEQLKGVSREDSLQKILDLADKKVSAEEFKELAKRKNDNYVKMIQDVSPADVYPGILQLLKDLRSNKIKIALASASKNGPFLLERMNLTGYFDAIADPAEVAASKAAPDIFIAAAHAVGVAPSESIGLEDSQAGIQAIKDSGALPIGVGRPEDLGDDIVIPDTSHYTLEFLKEVWLQKQK

trans-A βPGM$_{D10N,P146A}$:βG16BP:MgT complex (BMRB 51988)

MFKAVLFDLNGVITDTAEYHFRAWKALAEEIGINGVDRQFNEQLKGVSREDSLQKILDLADKKVSAEEFKELAKRKNDNYVKMIQDVSPADVYPGILQLLKDLRSNKIKIALASASKNGPFLLERMNLTGYFDAIADPAEVAASKAAPDIFIAAAHAVGVAPSESIGLEDSQAGIQAIKDSGALPIGVGRPEDLGDDIVIPDTSHYTLEFLKEVWLQKQK

········10·························38····44······53·····································96··················117························145·····················170···

Supplementary Figure 8 | Locations of unassigned (missing) residues in the $^{1}H^{15}N$-TROSY spectra of substrate-free βPGM species and βPGM complexes. Such residues are broadened beyond detection due to intermediate exchange on the millisecond timescale. Missing residues are identified by coloured text, according to the βPGM structural regions that are defined in Fig. 7, Supplementary Fig. 5 and Supplementary Fig. 9. Positions of the D10N and P146A substitutions are highlighted in yellow.

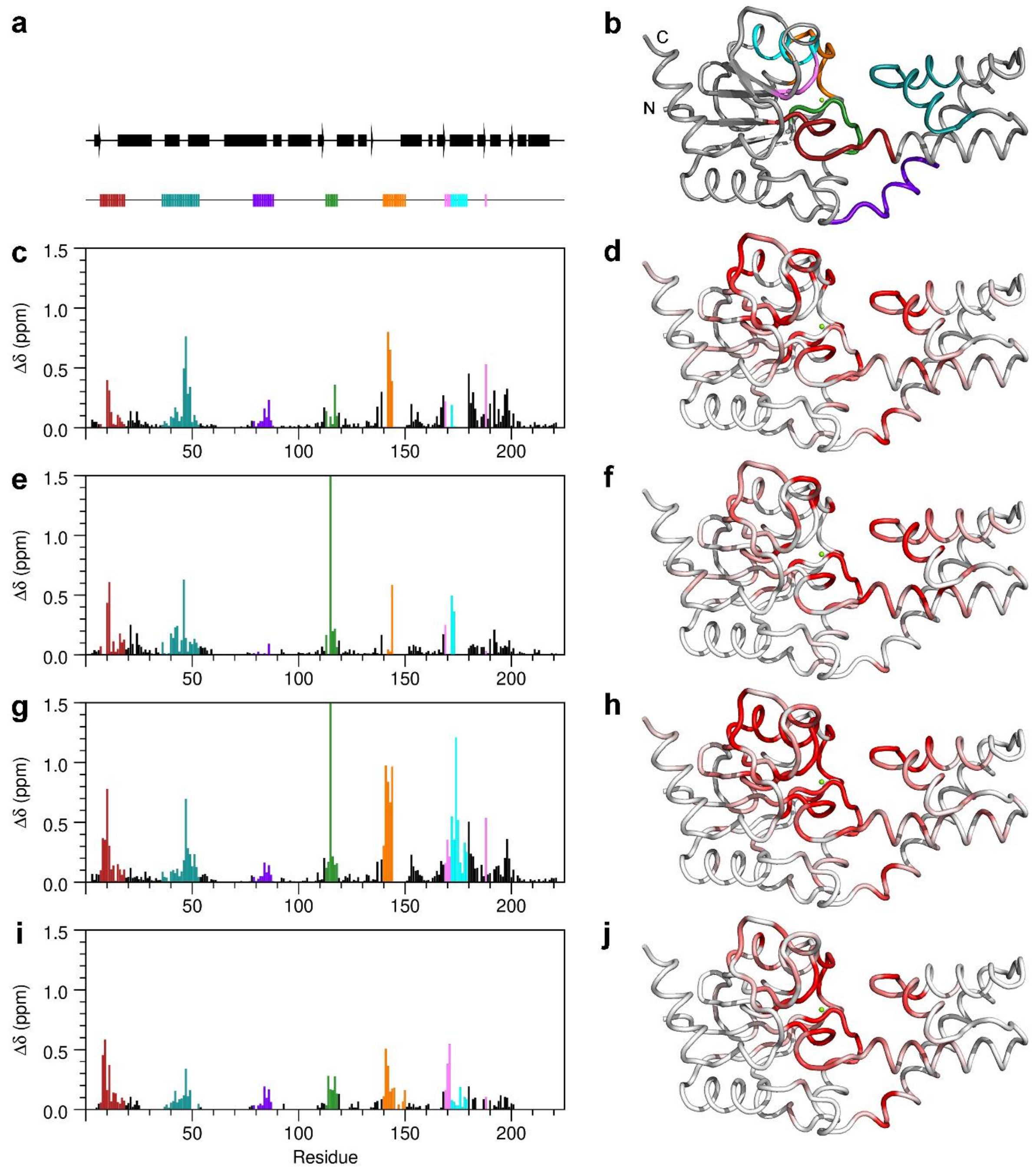

Supplementary Figure 9 | Weighted chemical shift perturbations reporting differences in the solution conformations of βPGM:βG16BP complexes. **a**, **b** Scheme showing the architecture of substrate-free *cis*-P βPGM_{WT} (PDB 2WHE[6]) with secondary structure elements indicated by bars (α-helices) and arrows (β-strands). Active site regions are highlighted by coloured bars and coloured cartoon backbone: general acid-base hinge (dark red, F7–E18), substrate specificity loop (teal, V36–L53), 80s hinge (purple, N79–S88), phosphodianions bridging loop (green, A113–N118), allomorphic control loop (orange, E140–I150), Mg_{cat} site (pink, E169–S171, V188) and 170s α-helix (cyan, Q172–K179). **c**–**h** Weighted chemical shift perturbations of the backbone amide group are calculated for each residue as: $\Delta\delta = [(\delta_{HN\text{-}X} - \delta_{HN\text{-}Y})^2 + (0.13 \times (\delta_{N\text{-}X} - \delta_{N\text{-}Y}))^2]^{1/2}$, where X and Y are the two βPGM complexes being compared. Δδ values are shown by coloured histogram bars and red-shaded cartoon

backbone. **c**, **d** Δδ values between the *cis*-A βPGM$_{D10N,P146A}$:βG16BP complex (BMRB 51989) and the *trans*-A βPGM$_{D10N,P146A}$:βG16BP complex (BMRB 51990). The small Δδ values for residues of the 80s hinge report that the *cis*-A βPGM$_{D10N,P146A}$:βG16BP complex adopts a NAC III conformation, whereas the *trans*-A βPGM$_{D10N,P146A}$:βG16BP complex adopts a NAC III^t conformation. This differential twist of the cap domain relative to the core domain is due to the different isomerisation state of the K145-A146 peptide bond in each complex. Further perturbation throughout the active site results in large Δδ values for non-exchange broadened residues comprising the general acid-base hinge, the substrate specificity loop, the phosphodianions bridging loop, the allomorphic control loop and in the vicinity of the Mg$_{cat}$ site. **e**, **f** Δδ values between the *trans*-A βPGM$_{D10N,P146A}$:βG16BP:MgT complex (BMRB 51988) and the *trans*-A βPGM$_{D10N,P146A}$:βG16BP complex (BMRB 51990). Almost negligible Δδ values for residues of the 80s hinge indicate that both complexes adopt the same NAC III^t conformation. For non-exchange broadened residues, large Δδ values in the active site loops report on the perturbations generated due to the occupancy of MgT. For A115, Δδ = 2.39 ppm, and has been truncated for clarity. **g**, **h** Δδ values between the *cis*-A βPGM$_{D10N,P146A}$:βG16BP complex (BMRB 51989) and the *trans*-A βPGM$_{D10N,P146A}$:βG16BP:MgT complex (BMRB 51988). The small Δδ values for residues of the 80s hinge report that the *cis*-A βPGM$_{D10N,P146A}$:βG16BP complex adopts a NAC III conformation, whereas the *trans*-A βPGM$_{D10N,P146A}$:βG16BP:MgT complex adopts a NAC III^t conformation. This differential twist of the cap domain relative to the core domain is due to the different isomerisation state of the K145-A146 peptide bond in each complex. Further perturbation throughout the active site along with occupancy of MgT results in large Δδ values for non-exchange broadened residues comprising the general acid-base hinge, the substrate specificity loop, the phosphodianions bridging loop, the allomorphic control loop and in the vicinity of the Mg$_{cat}$ site. For A115, Δδ = 2.48 ppm, and has been truncated for clarity. **i**, **j** Δδ values between the *cis*-P βPGM$_{D10N}$:βG16BP complex (BMRB 27174[5]) and the *cis*-P Mg$_{cat}$-free βPGM$_{D10N}$:βG16BP complex (BMRB 27175[5]). The Mg$_{cat}$ binding constant for this complex (K_d (Mg$_{cat}$) = 7.1 mM) mirrors the Mg^{2+} concentration in *L. lactis* cytoplasm (2–5 mM[8,9]). The small Δδ values observed for the 80s hinge report that the *cis*-P βPGM$_{D10N}$:βG16BP complex adopts a NAC III conformation, whereas the *cis*-P Mg$_{cat}$-free βPGM$_{D10N}$:βG16BP complex adopts a NAC III^t conformation. Here, the loss of Mg$_{cat}$ results in a twist of the cap domain relative to the core domain, while still maintaining a *cis* K145-P146 peptide bond. Pronounced Δδ values are clustered into discrete regions comprising the general acid-base hinge, the substrate specificity loop, the phosphodianions bridging loop, the allomorphic control loop and in the vicinity of the Mg$_{cat}$ site.

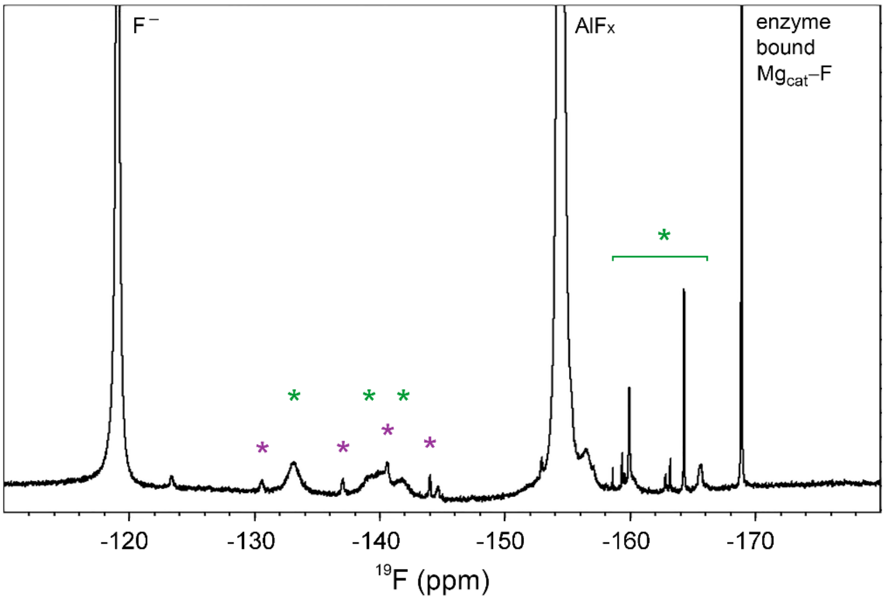

Supplementary Figure 10 | The βPGM$_{WT}$:AlF$_4$:F6P complex exists as a mixture of species. A ^{19}F NMR spectrum of a sample containing 0.5 mM βPGM$_{WT}$ in standard NMR buffer (50 mM K^+ HEPES (pH 7.2), 5 mM $MgCl_2$, 2 mM NaN_3, 10 % v/v ^2H_2O and 1 mM TSP) supplemented with 15 mM NaF, 5 mM $AlCl_3$ and 10 mM F6P was recorded using a Bruker 500 MHz Avance III HD spectrometer. The βPGM$_{WT}$:AlF$_4$:F6P complex populates several species with the four ^{19}F nuclei within each AlF_4^- moiety resonating at –133 ppm, –140 ppm, –142 ppm, and ranging between –159 and –133 ppm (green asterisks). The *cis* or *trans* isomerisation state of the K145-P146 peptide bond within each complex, together with or without binding of MgT may account for some of these species. A low population of a *cis*-P βPGM$_{WT}$:AlF$_4$:G6P complex with the four ^{19}F nuclei resonating at –131 ppm, –137 ppm, –141 ppm and –144 ppm (purple asterisks) is also observed[10]. The peaks for free F^- (–119 ppm), free AlFx (–154 ppm) and an enzyme-bound Mg$_{cat}$-F moiety (–169 ppm) have been truncated for clarity. The ^{19}F NMR spectrum of an equivalent sample recorded in the absence of $AlCl_3$ only shows a low population of a βPGM$_{WT}$:MgF$_3$:G6P complex and there is no clear evidence for a βPGM$_{WT}$:MgF$_3$:F6P complex.

Supplementary Table 1 | The interdomain hinge closure angle (º) describing cap domain movement with respect to the core domain for pairs of βPGM crystal structures determined using DynDom[11].

	1ZOL[a]	2WHE[a]	2WF9[b]	8Q1D[b]	8Q1E[b]	5OK1[c]	8Q1F[c,d]	8Q1F[c,e]	2WF5[f]	6YDJ[f]
1ZOL[a]	–	0.0	25.3	23.7	22.1	33.7	29.9	28.3	32.8	33.1
2WHE[a]	0.0	–	27.4	26.2	24.1	35.9	32.1	30.3	35.0	35.3

[a] Open conformation: substrate-free *cis*-P βPGM_{WT} (PDB 1ZOL[12], PDB 2WHE[6]). [b] NAC I conformation (typical range = 22.1–27.4°): *cis*-P βPGM_{WT}:BeF_3:G6P complex (PDB 2WF9[7]), *cis*-P βPGM_{D10N}:F16BP complex (PDB 8Q1D) and *trans*-A $\beta PGM_{D10N,P146A}$:F16BP:MgT complex (PDB 8Q1E). [c] NAC III conformation (typical range = 28.3–35.9°): *cis*-P βPGM_{D10N}:βG16BP complex (PDB 5OK1[5]), *trans*-A $\beta PGM_{D10N,P146A}$:βG16BP:MgT complex (PDB 8Q1F, [d] chain A, [e] chain B). [f] Fully closed near-transition state conformation (typical range = 32.8–35.3°): *cis*-P βPGM_{WT}:MgF_3:G6P complex (PDB 2WF5[6]) and *cis*-A βPGM_{P146A}:MgF_3:G6P complex (PDB 6YDJ[2]).

Supplementary Note 1 | During the catalytic cycle of βPGM, the carboxylate sidechain of the general acid-base undergoes a positional switch between a solvent exposed location and one within the active site, where it facilitates proton transfer to or from the different nascent leaving groups[5,13]. As an acid, D10 delivers a proton to βG16BP during phosphoryl transfer from βG16BP to βPGM, while as a base it accepts a proton from either G6P or βG1P during phosphoryl transfer from βPGM^{P} to G6P/βG1P[14] (Supplementary Fig. 1a). When functioning as an acid, protonation of the carboxylate sidechain may arise passively via a neighbouring water molecule, but this would be against a substantial pK_a gradient. Instead, in the *cis*-P βPGM_{D10N}:F16BP complex (PDB 8Q1D), the close proximity of the sidechains of D8 and D170, within an anion-rich environment, illustrates that one of these carboxylate oxygen atoms may be protonated (Fig. 4a, Fig. 5a). A rotation of the sidechain of D170 could deliver this proton to the sidechain of D10 and hence the nascent leaving group, via a water molecule coordinating Mg_{cat}, during the structural transition from the NAC I conformation to the NAC III conformation (Supplementary Fig. 3). Evidence to support the existence of such a proton shuttling pathway is provided by the catalytic behaviour of βPGM_{D170N}. Here, the carboxamide group of N170 interrupts the pathway and not only leads to a marked retardation of overall turnover compared with βPGM_{WT} but also produces a substantial accumulation of βG16BP in solution[15], as D10 remains effective as a base but its role is diminished as an acid.

Supplementary Note 2 | Inspection of the NMR spectra acquired for resonance assignment of the substrate-free βPGM species and βPGM complexes revealed that intermediate conformational exchange on the millisecond timescale was occurring for a selection of residues (Supplementary Fig. 8). In all of the substrate-free βPGM species, inherent dynamic exchange results in missing assignments for residues of the general acid-base hinge (L9, D10, G11), the substrate specificity loop (R38, L44, K45, G46, S48, R49, E50, D51, S52, L53), the phosphodianions bridging loop (K117, N118) and the Mg_{cat} site (D170, S171). These residues locate to the active site and it is likely that the absence of a bound substrate partly accounts for the observed millisecond exchange. Furthermore, the presence of a *trans* K145-P146 peptide bond introduces additional exchange broadening for residues of the allomorphic control loop (K145, A147, D149, I150) and the 170s α-helix, whereas a *trans* K145-A146 peptide bond suppresses this dynamic process. Distinct behaviour is also observed on D10 to N10 substitution of the residue assigned as the general acid-base, where the carboxamide sidechain of N10 mimics a protonated carboxylate sidechain of D10. Twelve contiguous residues of the general acid-base hinge (F7–E18) are absent, together with some additional residues of the phosphodianions bridging loop. During the catalytic cycle, the sidechain of the general acid-base switches between a solvent exposed location and a position within the active site to facilitate proton transfer to the nascent leaving group[5,13]. In substrate-free βPGM, the carboxylate sidechain of D10 favours the rotated out arrangement, whereas the carboxamide sidechain of N10 leads to dynamic transitioning of the general acid-base residue into the active site.

On formation of the *cis*-P $βPGM_{D10N}$:F16BP complex (BMRB 51985), residues of the substrate specificity loop became assignable in the NMR spectra since the intermediate exchange dynamic is suppressed by F16BP bound in the active site (Supplementary Fig. 8). However, some of the inherent dynamics apparent in the substrate-free βPGM species still persist, along with the sidechain repositioning dynamic associated with N10. Residues of the general acid-base hinge (D8, L9, N10, G11, V12, I13, T14, D15), the phosphodianions bridging loop (A113, S114, A115, S116, K117), the allomorphic control loop (V141, S144, K145), the Mg_{cat} site (S171, Q172, V188) and 170s α-helix (G174) are missing and reflect the dynamic nature of the complex. As domain closure is strongly stimulated by the binding of a phosphodianion group of F16BP in the distal region of the active site[16], substantial interdomain hinge opening from a NAC I conformation is suppressed and the open conformation is unlikely to be a sizeable population within the conformational ensemble of the *cis*-P $βPGM_{D10N}$:F16BP complex. This interpretation is supported by the distribution of chemical shift behaviour of both I84 and S88, which act as reporters of the interdomain hinge closure angle. The *trans*-A $βPGM_{D10N,P146A}$:F16BP complex (BMRB 51987) retains the exchange process relating to the

phosphodianions bridging loop, but the repositioning dynamic associated with N10 is lost as most of the missing residues in the general acid-base hinge are assignable in the NMR spectra. Interestingly, several residues of the allomorphic control loop (K145, A146, A147, D149, I150) undergo intermediate exchange and are broadened beyond detection. In the substrate-free βPGM species, these residues are specifically associated with dynamics of a *trans* K145-P146 peptide bond, whereas in the *trans*-A $\beta PGM_{D10N,P146A}$:F16BP complex, this conformational exchange is occurring in a *trans* K145-A146 peptide bond background. This alteration away from the substrate-free $\beta PGM_{D10N,P146A}$ behaviour indicates that the occupancy of F16BP in a NAC I conformation is able to modulate the dynamic propensity of the allomorphic control loop. Furthermore, binding of MgT within the active site of the *trans*-A $\beta PGM_{D10N,P146A}$:F16BP:MgT complex (BMRB 51986) acts to suppress almost all of the intermediate exchange dynamics (only residue I150 is missing), which generates a trapped and dynamically restrained complex.

Dynamically restrained complexes are also observed for the *cis*-P βPGM_{D10N}:βG16BP complex (BMRB 27174[5]), the *cis*-P Mg_{cat}-free βPGM_{D10N}:βG16BP complex (BMRB 27175[5]) and the *cis*-A $\beta PGM_{D10N,P146A}$:βG16BP complex (BMRB 51989), which are all NAC III conformations. All backbone resonances in their respective $^{1}H^{15}N$-TROSY spectra have been assigned (Supplementary Fig. 8). Here, the stabilising properties of the *cis* K145-X146 peptide bond, together with the presence of βG16BP restrict the available dynamics even in the absence of Mg_{cat}. In contrast, substantial dynamics on the millisecond timescale occurs in the *trans*-A $\beta PGM_{D10N,P146A}$:βG16BP complex (BMRB 51990). Eighteen residues are broadened beyond detection and these locate to the general acid-base hinge (D8, L9), the phosphodianions bridging loop (S114), the allomorphic control loop (E140, V141, K145, A146, A147, D149, I150), the Mg_{cat} site (D170, S171) and the 170s α-helix (G174, I175, Q176, A177, I178, K179) and are related to the adoption of a *trans* K145-A146 peptide bond. Binding of MgT in the *trans*-A $\beta PGM_{D10N,P146A}$:βG16BP:MgT complex (BMRB 51988) fails to suppress completely the dynamics of the allomorphic control loop associated with a *trans* K145-A146 peptide bond, as five residues (K145, A146, A147, D149, I150) are absent from the NMR spectra due to intermediate exchange broadening. This observation implies that the occupancy of βG16BP in a NAC III conformation is able to stimulate dynamic exchange in residues of the allomorphic control loop, consistent with this loop accessing conformers associated with both *trans* and *cis* forms of the K145-A146 peptide bond.

Supplementary References

1. Dai, J., Wang, L., Allen, K. N., Rådström, P. & Dunaway-Mariano, D. Conformational cycling in β-phosphoglucomutase catalysis: Reorientation of the β-D-glucose 1,6-(bis)phosphate intermediate. *Biochemistry* **45**, 7818–7824 (2006).

2. Wood, H. P., Cruz-Navarrete, F. A., Baxter, N. J., Trevitt, C. R., Robertson, A. J., Dix, S. R., Hounslow, A. M., Cliff, M. J. & Waltho, J. P. Allomorphy as a mechanism of post-translational control of enzyme activity. *Nat. Commun.* **11**, 1–12 (2020).

3. Midelfort, C. F., Gupta, R. K. & Rose, I. A. Fructose 1,6-bisphosphate: Isomeric composition, kinetics, and substrate specificity for the aldolases. *Biochemistry* **15**, 2178–2185 (1976).

4. Cruz-Navarrete, F. A., Baxter, N. J., Wood, H. P., Hounslow, A. M. & Waltho, J. P. ^{1}H, ^{15}N and ^{13}C backbone resonance assignment of the P146A variant of β-phosphoglucomutase from *Lactococcus lactis* in its substrate-free form. *Biomol. NMR Assign.* **13**, 349–356 (2019).

5. Johnson, L. A., Robertson, A. J., Baxter, N. J., Trevitt, C. R., Bisson, C., Jin, Y.; Wood, H. P., Hounslow, A. M., Cliff, M. J., Blackburn, G. M., Bowler, M. W. & Waltho, J. P. van der Waals contact between nucleophile and transferring phosphorus is insufficient to achieve enzyme transition-state architecture. *ACS Catal.* **8**, 8140–8153 (2018).

6. Baxter, N. J., Bowler, M. W., Alizadeh, T., Cliff, M. J., Hounslow, A. M., Wu, B., Berkowitz, D. B., Williams, N. H., Blackburn, G. M. & Waltho, J. P. Atomic details of near-transition state conformers for enzyme phosphoryl transfer revealed by MgF_3^- rather than by phosphoranes. *Proc. Natl Acad. Sci. USA* **107**, 4555–4560 (2010).

7. Griffin, J. L., Bowler, M. W., Baxter, N. J., Leigh, K. N., Dannatt, H. R. W., Hounslow, A. M., Blackburn, G. M., Webster, C. E., Cliff, M. J. & Waltho, J. P. Near attack conformers dominate

β-phosphoglucomutase complexes where geometry and charge distribution reflect those of substrate. *Proc. Natl Acad. Sci. USA* **109**, 6910–6915 (2012).

8. Goel, A., Santos, F., de Vos, W. M., Teusink, B. & Molenaar, D. Standardized assay medium to measure *Lactococcus lactis* enzyme activities while mimicking intracellular conditions. *Appl. Environ. Microbiol.* **78**, 134–143 (2012).

9. Zamberlin, Š., Antunac, N., Havranek, J. & Samaržija, D. Mineral elements in milk and dairy products. *Mljekarstvo* **62**, 111–125 (2012).

10. Baxter, N. J., Blackburn, G. M., Marston, J. P., Hounslow, A. M., Cliff, M. J., Bermel, W., Williams, N. H., Hollfelder, F., Wemmer, D. E. & Waltho, J. P. Anionic charge is prioritized over geometry in aluminum and magnesium fluoride transition state analogs of phosphoryl transfer enzymes. *J. Am. Chem. Soc.* **130**, 3952–3958 (2008).

11. Hayward, S. & Berendsen, H. J. C. Systematic analysis of domain motions in proteins from conformational change: New results on citrate synthase and T4 lysozyme. *Proteins: Struct., Funct., and Bioinf.* **30**, 144–154 (1998).

12. Zhang, G., Dai, J., Wang, L., Dunaway-Mariano, D., Tremblay, L. W. & Allen, K. N. Catalytic cycling in β-phosphoglucomutase: A kinetic and structural analysis. *Biochemistry* **44**, 9404–9416 (2005).

13. Dai, J., Finci, L., Zhang, C., Lahiri, S., Zhang, G., Peisach, E., Allen, K. N. & Dunaway-Mariano, D. Analysis of the structural determinants underlying discrimination between substrate and solvent in β-phosphoglucomutase catalysis. *Biochemistry* **48**, 1984–1995 (2009).

14. Robertson, A. J., Wilson, A. L., Burn, M. J., Cliff, M. J., Popelier, P. L. & Waltho, J. P. The relationship between enzyme conformational change, proton transfer, and phosphoryl transfer in β-phosphoglucomutase. *ACS Catal.* **11**, 12840–12849 (2021).

15. Wood, H. P., Baxter, N. J., Cruz-Navarrete, F. A., Trevitt, C. R., Hounslow, A. M. & Waltho, J. P. Enzymatic production of β-glucose 1,6-bisphosphate through manipulation of catalytic magnesium coordination. *Green Chem.* **23**, 752–762 (2021).

16. Robertson, A. J., Cruz-Navarrete, F. A., Wood, H. P., Vekaria, N., Hounslow, A. M., Bisson, C., Cliff, M. J., Baxter, N. J. & Waltho, J. P. An enzyme with high catalytic proficiency utilizes distal site substrate binding energy to stabilize the closed state but at the expense of substrate inhibition. *ACS Catal.* **12**, 3149–3164 (2022).
