# Supplementary material for: Peri active site catalysis of proline isomerisation is the molecular basis of allomorphy in β-phosphoglucomutase
Source: Commun Biol. 2024 Jul 27;7:909. doi: 10.1038/s42003-024-06577-9 (PMC11283535; doi:10.1038/s42003-024-06577-9)
Supplement: Supplementary file 3 — Description of Additional Supplementary Materials [file 42003_2024_6577_MOESM3_ESM.docx]

**Description of Additional Supplementary Files**

**File name:** Supplementary Data 1

**Description:** Intrinsic Euler angles (º) used to create Figure 6.

**File name:** Supplementary Data 2

**Description:** Legend and ^1^H^15^N-TROSY chemical shift comparisons of substrate-free βPGM species and βPGM complexes.

**File name:** Supplementary Data 3

**Description:** ^1^HN and ^15^N chemical shift data (ppm) used to create Supplementary Data 2, Figure 3, Figure 7, Supplementary Figure 5 and Supplementary Figure 9.

**File name:** Supplementary Data 4

**Description:** ^1^HN and ^15^N chemical shift data (ppm) used to create Supplementary Figure 5 and Supplementary Figure 6.
