# Supplementary material for: Peri active site catalysis of proline isomerisation is the molecular basis of allomorphy in β-phosphoglucomutase
Source: Commun Biol. 2024 Jul 27;7:909. doi: 10.1038/s42003-024-06577-9 (PMC11283535; doi:10.1038/s42003-024-06577-9)
Supplement: Supplementary file 5 — Supplementary Data 2 [file 42003_2024_6577_MOESM5_ESM.pdf]

Supplementary Data 2 | Legend and $^{1}H^{15}N$-TROSY chemical shift comparisons of substrate-free βPGM species and βPGM complexes. Backbone amide ^{1}HN chemical shifts (x-axis with reversed sense and range = 3 ppm) and backbone amide ^{15}N chemical shifts (y-axis with reversed sense and range = 12 ppm) derived from $^{1}H^{15}N$-TROSY spectra are plotted for each residue of the substrate-free βPGM species and βPGM complexes. Most of the chemical shift values have been deposited in the BMRB and all are provided in Supplementary Data 3. Symbols are coloured according to the interdomain hinge closure angle as: open conformation (black), NAC I conformation (gold), NAC III conformation (pink and purple) and the fully closed near-transition state conformation (blue).

Symbol	βPGM species or complex	Accession number
○	Substrate-free *cis*-P βPGM$_{WT}$	BMRB 28095[1]
□	Substrate-free *trans*-P βPGM$_{WT}$	BMRB 28096[1]
◇	Substrate-free *trans*-A βPGM$_{P146A}$	BMRB 27920[2]
○	Substrate-free *cis*-P βPGM$_{D10N}$	
□	Substrate-free *trans*-P βPGM$_{D10N}$	
◇	Substrate-free *trans*-A βPGM$_{D10N,P146A}$	
○	*cis*-P βPGM$_{D10N}$:F16BP complex	BMRB 51985
◇	*trans*-A βPGM$_{D10N,P146A}$:F16BP:MgT complex	BMRB 51986
◇	*trans*-A βPGM$_{D10N,P146A}$:F16BP complex	BMRB 51987
○	*cis*-P βPGM$_{D10N}$:βG16BP complex	BMRB 27174[3]
○	*cis*-P Mg$_{cat}$-free βPGM$_{D10N}$:βG16BP complex	BMRB 27175[3]
◇	*trans*-A βPGM$_{D10N,P146A}$:βG16BP:MgT complex	BMRB 51988
○	*cis*-A βPGM$_{D10N,P146A}$:βG16BP complex	BMRB 51989
◇	*trans*-A βPGM$_{D10N,P146A}$:βG16BP complex	BMRB 51990
○	*cis*-P βPGM$_{WT}$:MgF$_3$:G6P complex	BMRB 7234[4]
○	*cis*-A βPGM$_{P146A}$:MgF$_3$:G6P complex	BMRB 28097[1]

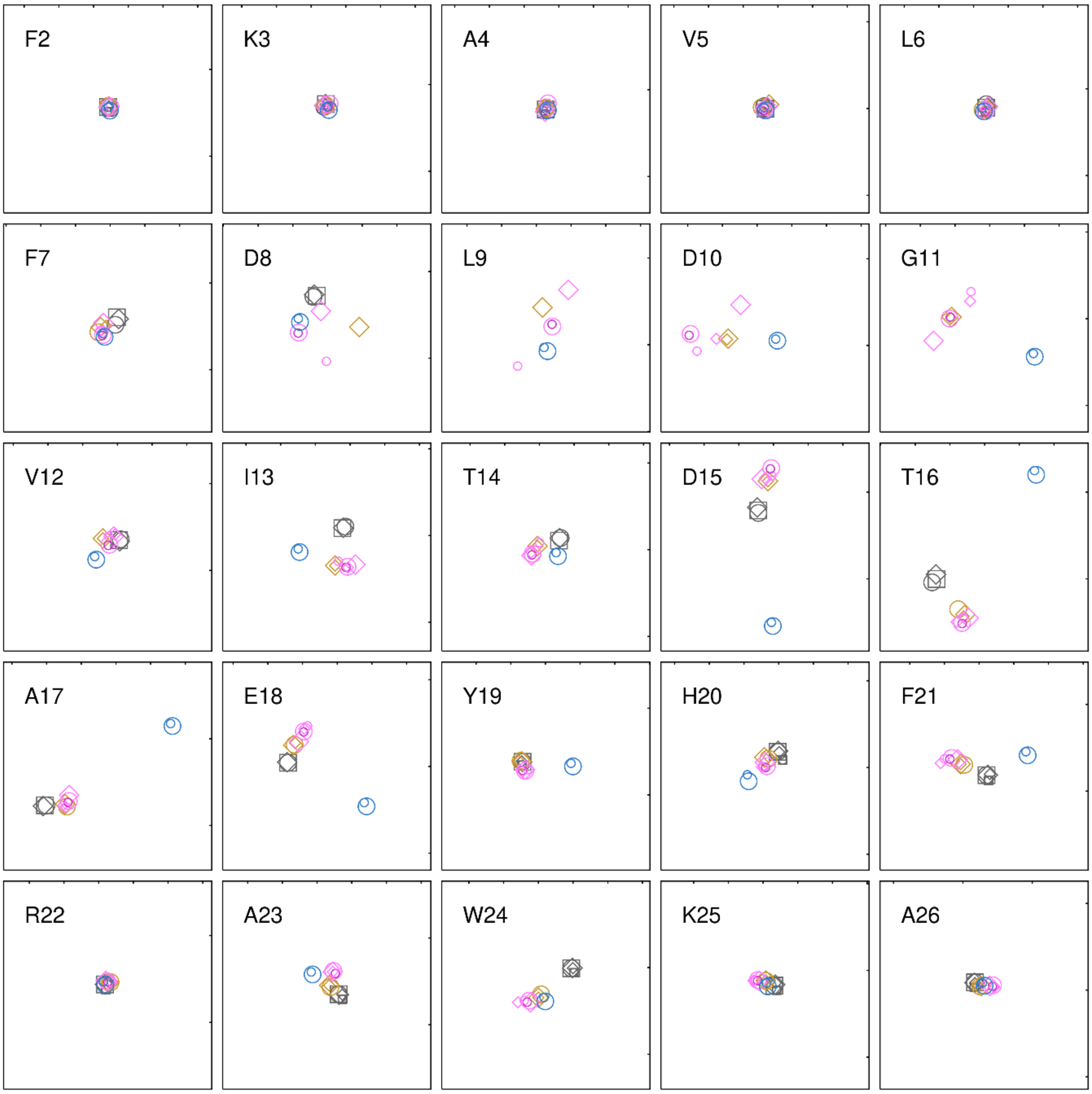

Supplementary Data 2 – continued

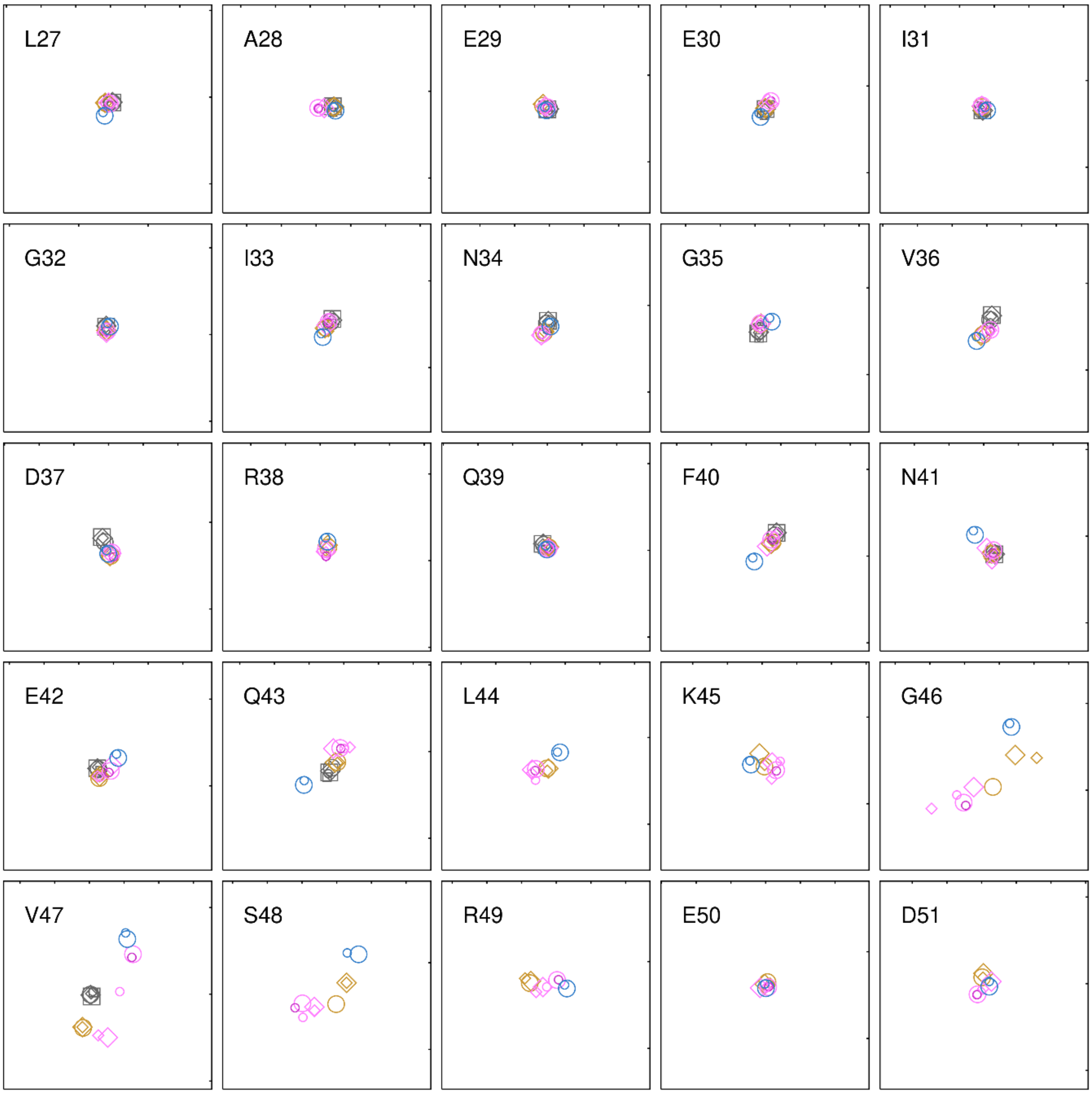

Supplementary Data 2 – continued

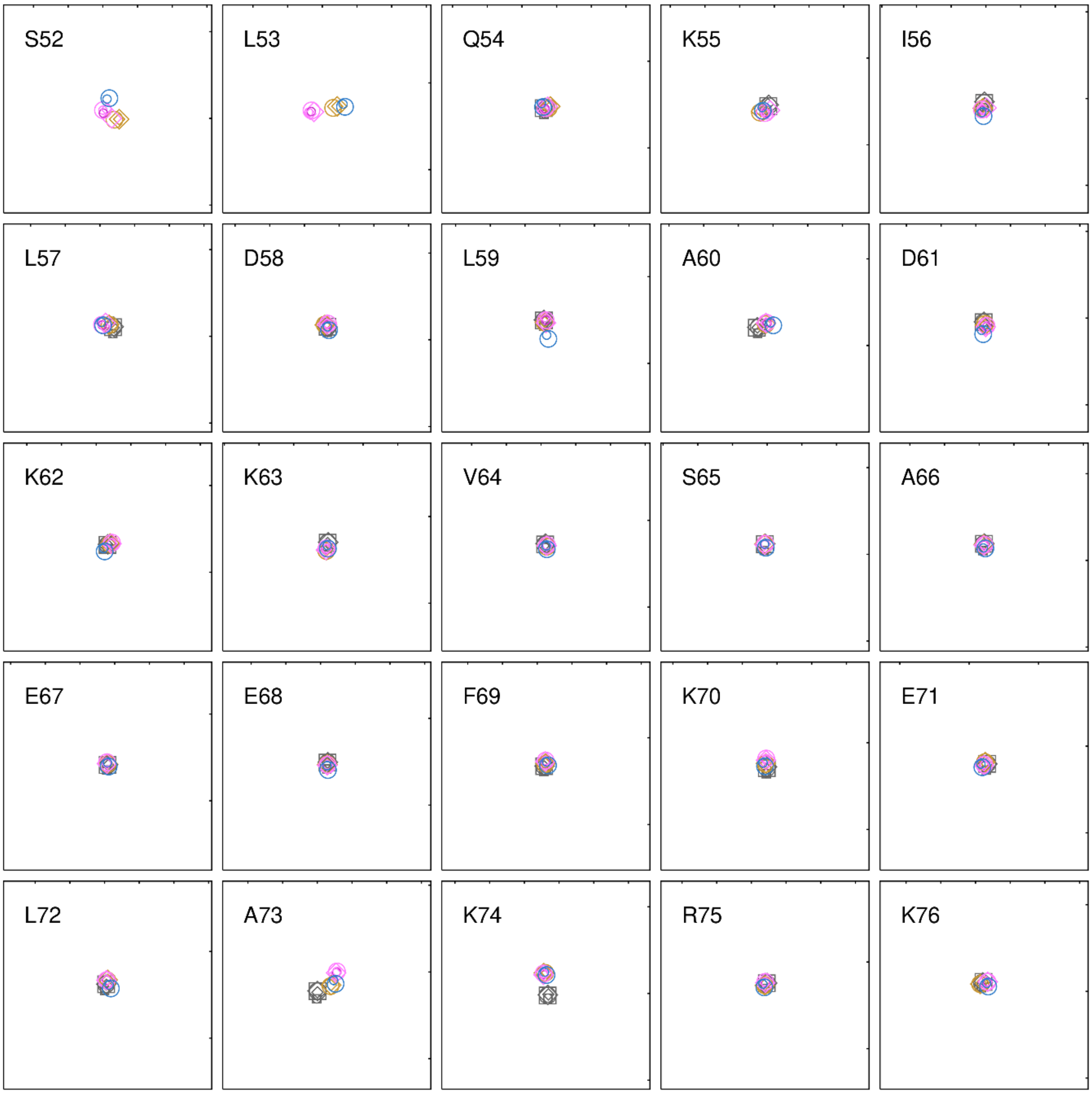

Supplementary Data 2 – continued

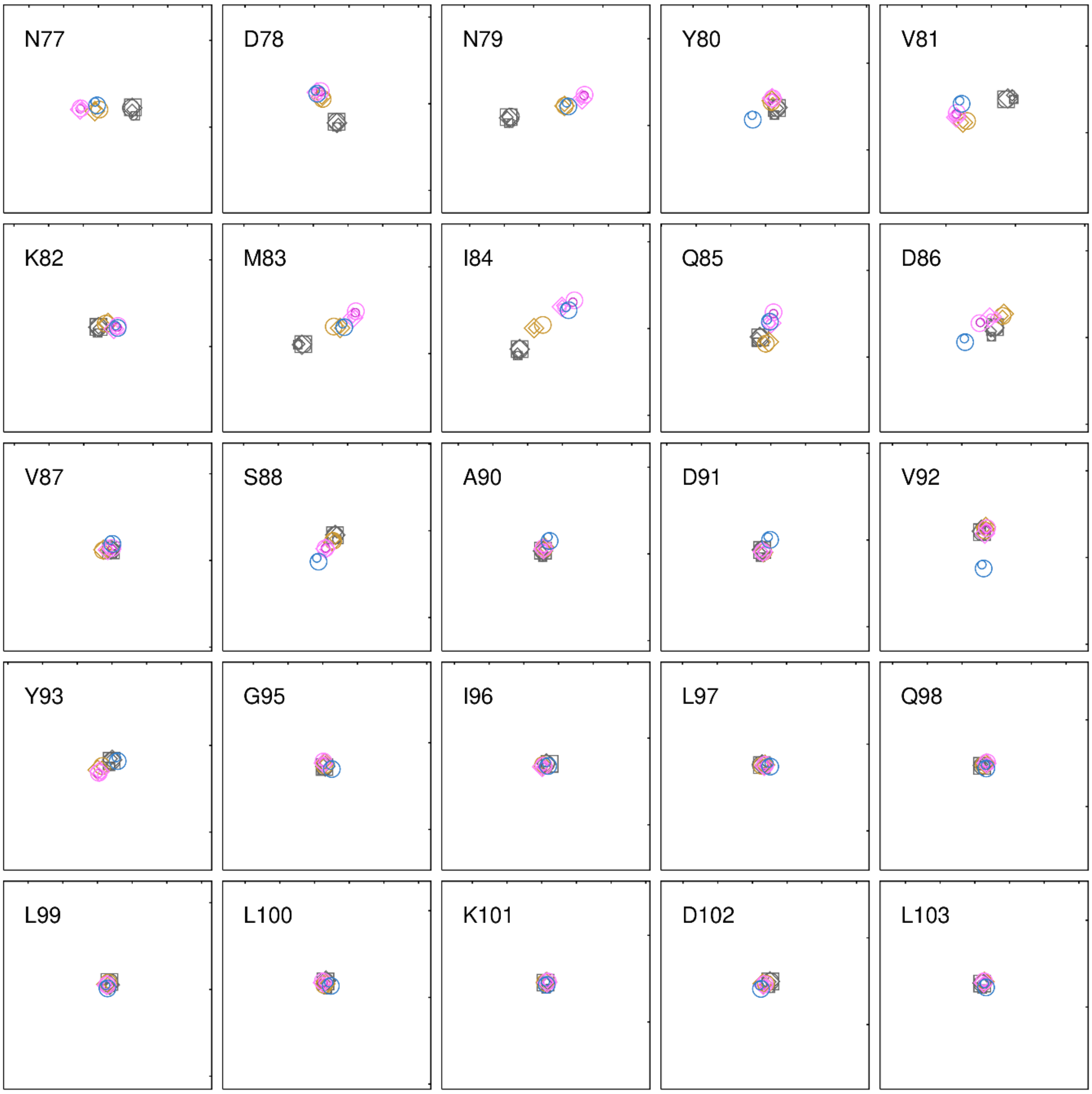

Supplementary Data 2 – continued

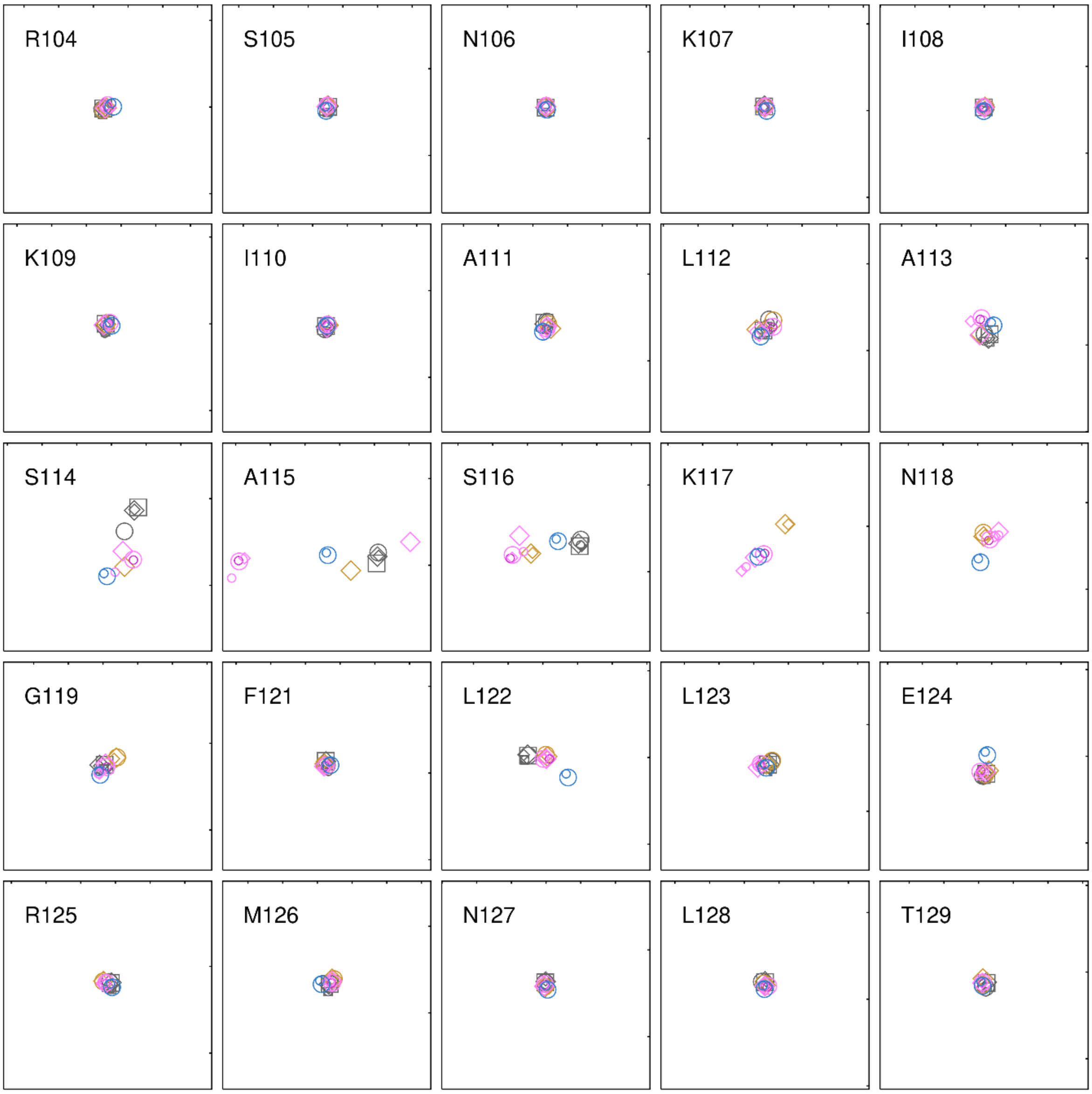

Supplementary Data 2 – continued

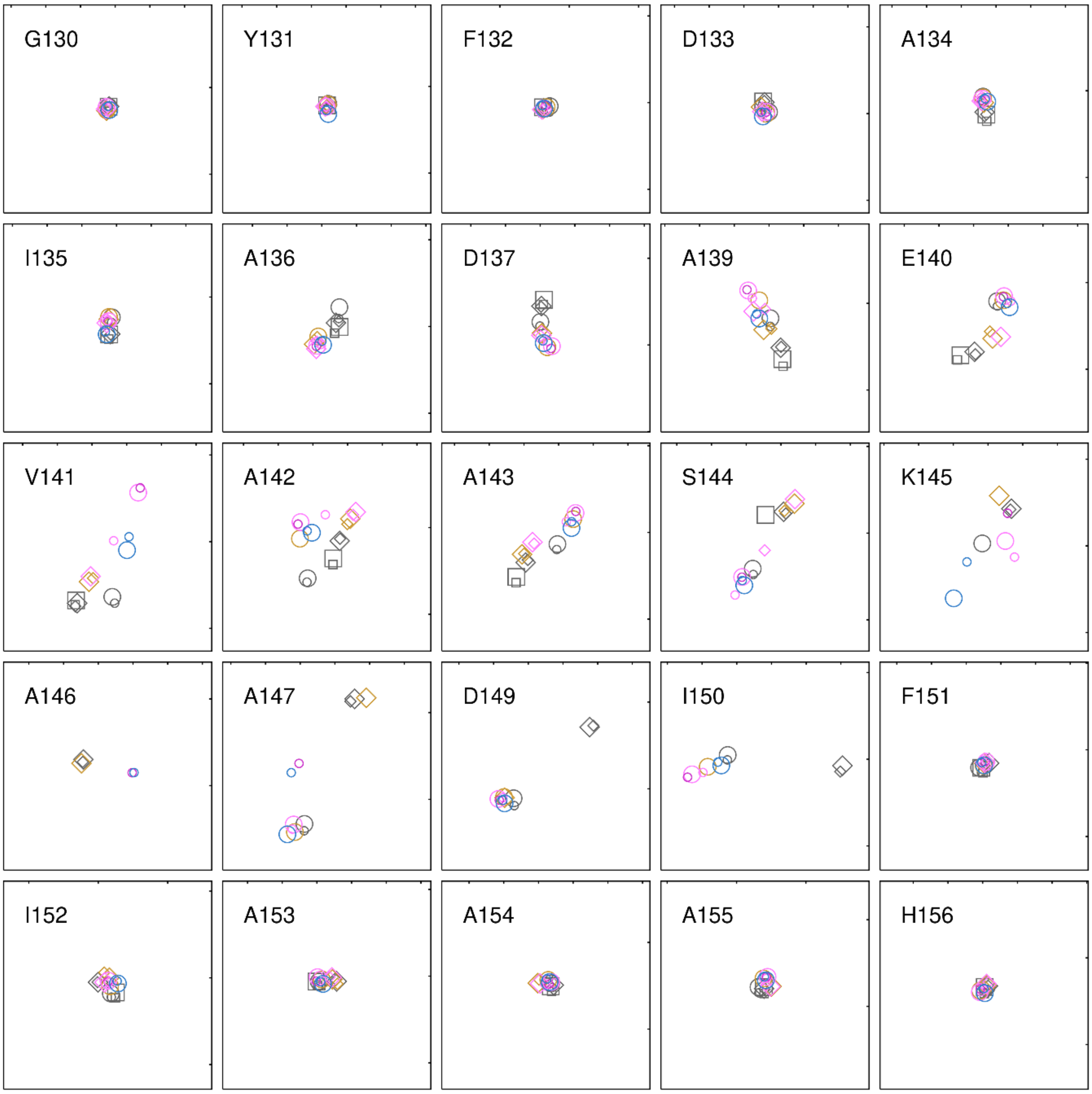

Supplementary Data 2 – continued

For many residues of the allomorphic control loop, the population of either a *trans* K145-X146 peptide bond or a *cis* K145-X146 peptide bond results in diagnostic $^{1}H^{15}N$-TROSY chemical shift behaviour. A comparison of structures containing a *trans* K145-A146 peptide bond (PDB 8Q1E, PDB 8Q1F, PDB 6YDK[1]) with those containing a *cis* K145-X146 peptide bond (PDB 8Q1D, PDB 2WHE[4], PDB 2WF9[5], PDB 5OK1[3], PDB 2WF5[4], PDB 6YDJ[1]) reveals that there is a clear correlation with the identity of the hydrogen bond acceptor in the vicinity of the amide group of I150. In the *trans*-A complexes, where the alkylammonium sidechain of K145 is exposed to solvent, the hydrogen bond acceptor is the carbonyl group of A142. However, in the *cis*-X complexes, where the alkylammonium sidechain is instead engaged in the active site, the hydrogen bond acceptor is the carbonyl group of A143. Here, this change in register and reorganisation of the backbone of the allomorphic control loop is mirrored in solution, which occurs as a direct result of the isomerisation state of the K145-X146 peptide bond and is independent of the interdomain hinge closure angle.

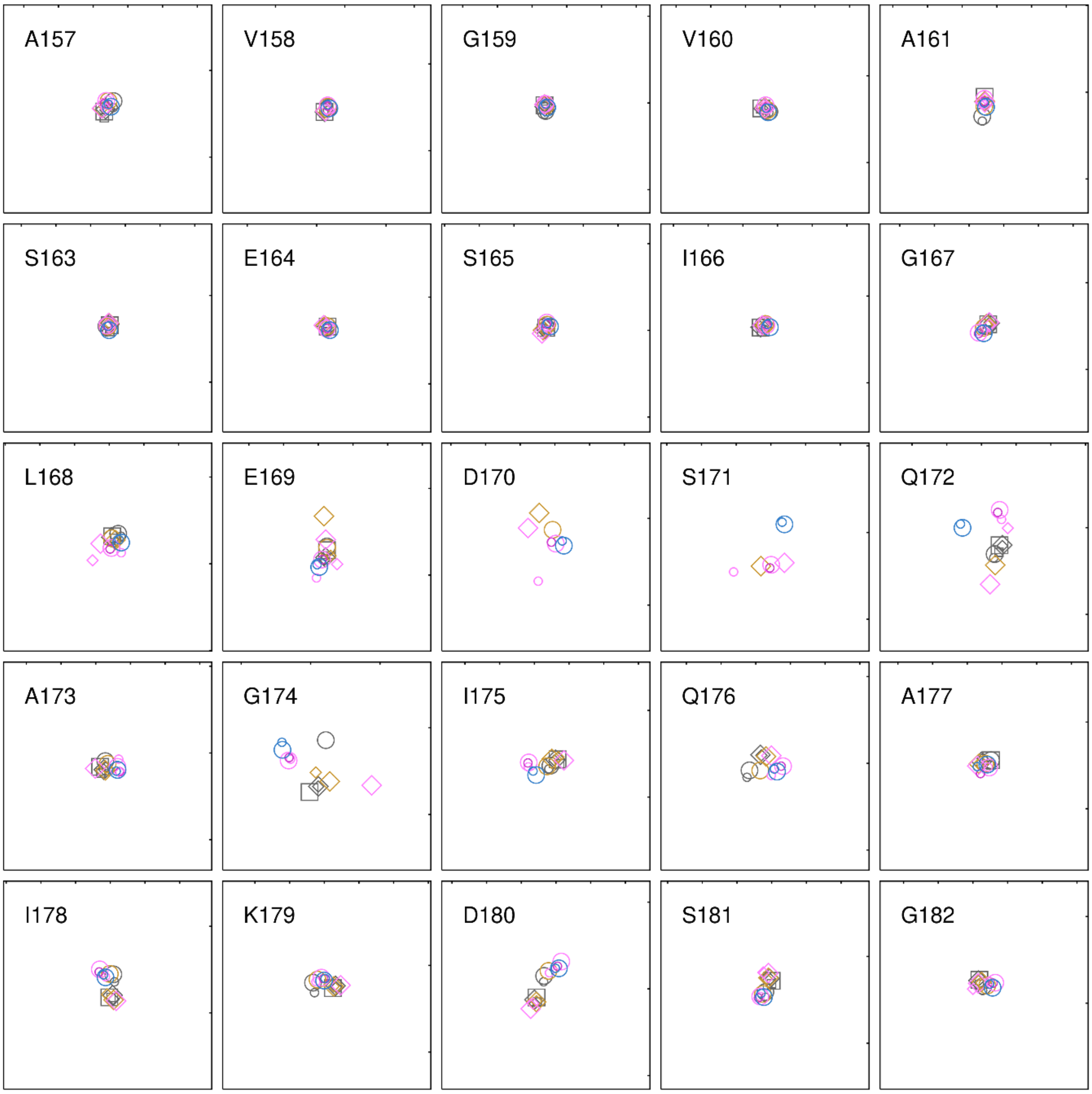

Supplementary Data 2 – continued

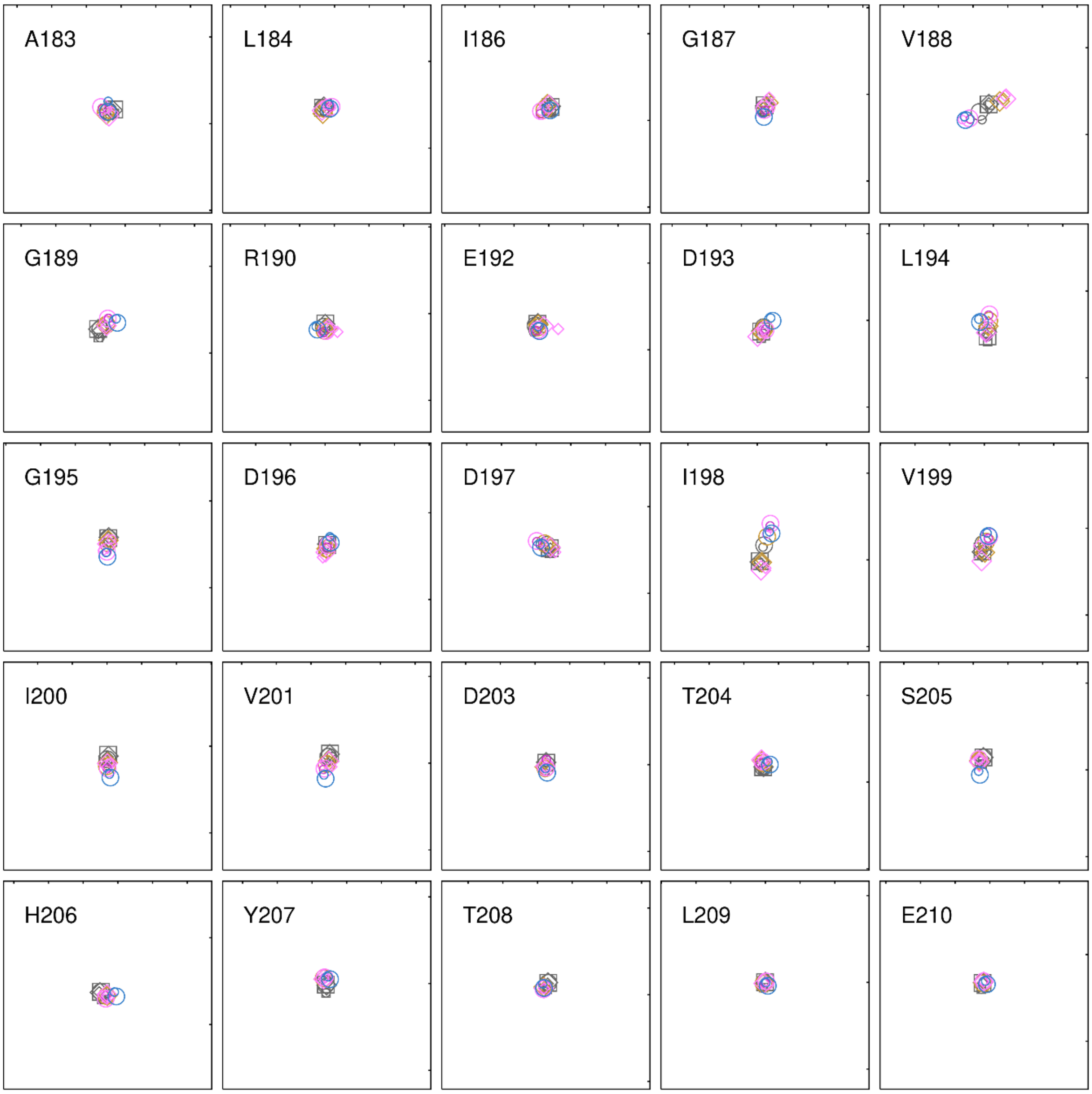

Supplementary Data 2 – continued

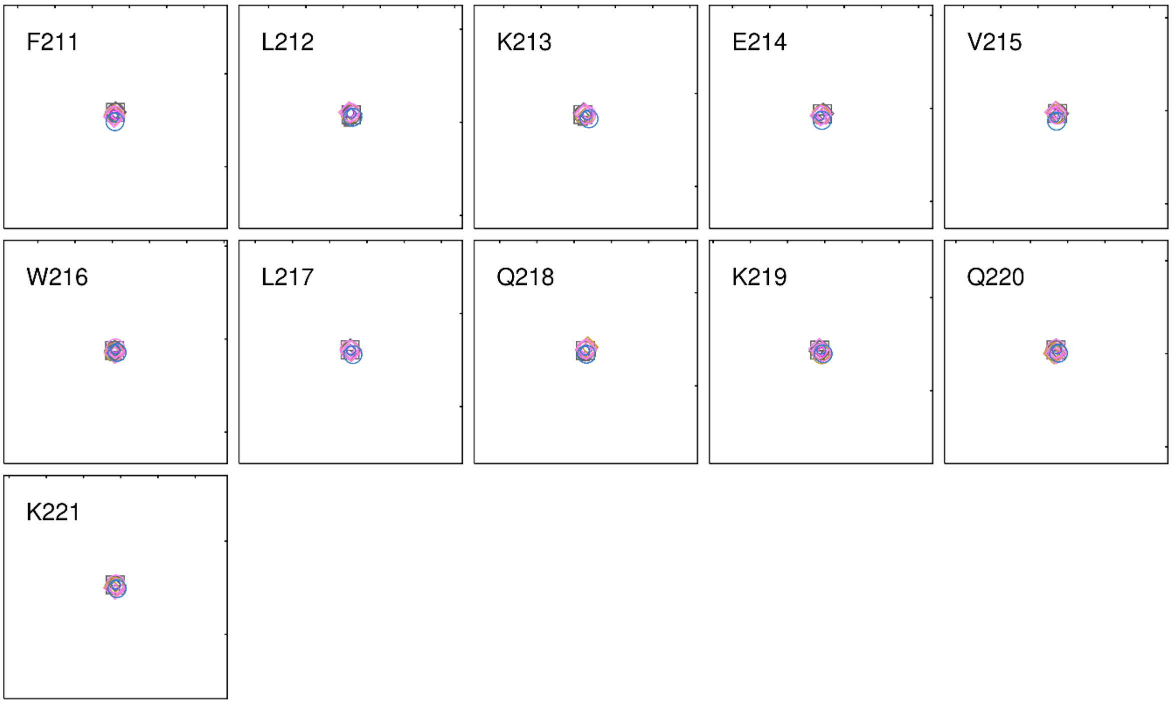

Supplementary Data 2 – continued

Supplementary Data 2 References

1. Wood, H. P., Cruz-Navarrete, F. A., Baxter, N. J., Trevitt, C. R., Robertson, A. J., Dix, S. R., Hounslow, A. M., Cliff, M. J. & Waltho, J. P. Allomorphy as a mechanism of post-translational control of enzyme activity. *Nat. Commun.* **11**, 1–12 (2020).

2. Cruz-Navarrete, F. A., Baxter, N. J., Wood, H. P., Hounslow, A. M. & Waltho, J. P. ^{1}H, ^{15}N and ^{13}C backbone resonance assignment of the P146A variant of β-phosphoglucomutase from *Lactococcus lactis* in its substrate-free form. *Biomol. NMR Assign.* **13**, 349–356 (2019).

3. Johnson, L. A., Robertson, A. J., Baxter, N. J., Trevitt, C. R., Bisson, C., Jin, Y.; Wood, H. P., Hounslow, A. M., Cliff, M. J., Blackburn, G. M., Bowler, M. W. & Waltho, J. P. van der Waals contact between nucleophile and transferring phosphorus is insufficient to achieve enzyme transition-state architecture. *ACS Catal.* **8**, 8140–8153 (2018).

4. Baxter, N. J., Bowler, M. W., Alizadeh, T., Cliff, M. J., Hounslow, A. M., Wu, B., Berkowitz, D. B., Williams, N. H., Blackburn, G. M. & Waltho, J. P. Atomic details of near-transition state conformers for enzyme phosphoryl transfer revealed by MgF_3^- rather than by phosphoranes. *Proc. Natl Acad. Sci. USA* **107**, 4555–4560 (2010).

5. Griffin, J. L., Bowler, M. W., Baxter, N. J., Leigh, K. N., Dannatt, H. R. W., Hounslow, A. M., Blackburn, G. M., Webster, C. E., Cliff, M. J. & Waltho, J. P. Near attack conformers dominate β-phosphoglucomutase complexes where geometry and charge distribution reflect those of substrate. *Proc. Natl Acad. Sci. USA* **109**, 6910–6915 (2012).
